# Paradoxical Effect of Obesity on Survival Outcomes in Prostate Cancer Patients Receiving Enzalutamide and Abiraterone

**DOI:** 10.3390/medicina62010202

**Published:** 2026-01-18

**Authors:** Bahattin Engin Kaya, Mehmet Zahid Koçak, Oğuzhan Yıldız, Talat Aykut, Ali Fuat Gürbüz, Ömer Genç, Melek Karakurt Eryılmaz, Murat Araz, Mehmet Artaç

**Affiliations:** Department of Medical Oncology, Necmettin Erbakan University School of Medicine, Konya 42080, Turkey; mehmetzahidkocak@hotmail.com (M.Z.K.); dr.oguzhan@outlook.com (O.Y.); talat_aykut@hotmail.com (T.A.); dr.alifuatgurbuz@hotmail.com (A.F.G.); omergenc58@hotmail.com (Ö.G.); drangelkarakurt@hotmail.com (M.K.E.); zaratarum@yahoo.com (M.A.); mehmetartac@yahoo.com (M.A.)

**Keywords:** body mass index, obesity paradox, androgen receptor inhibitors, castration-resistant prostate cancer, castration-sensitive prostate cancer, survival outcomes

## Abstract

*Background and Objectives*: Overweight and obesity is defined as a Body Mass Index (BMI) of 25.0–29.9 kg/m^2^ and ≥30.0 kg/m^2^. The prognostic significance of obesity in metastatic prostate cancer is still unclear, especially between the castration-sensitive (CSPC) and castration-resistant (CRPC) disease states. New evidence suggests that obese patients who get androgen receptor–targeted therapies may have an unexpected survival advantage. This study examined the correlation between body mass index (BMI) and survival outcomes in patients administered androgen receptor pathway inhibitors. *Materials and Methods*: A retrospective analysis was conducted on a cohort of 167 patients with metastatic prostate cancer treated between 2015 and 2024. BMI was analyzed as both a continuous variable and a categorical variable, which was classified as normal weight, overweight or obese. The primary goal of this study is to compare PFS and OS among BMI groups. We employed Kaplan–Meier survival analysis and Cox regression modeling to evaluate prognostic indicators. The CSPC and CRPC groups were evaluated separately. *Results*: PFSs for normal, overweight and obese CSPC patients were 11.3, 15.1, 19.5 months, respectively; *p* = 0.03 but the OS did not differ significantly between BMI groups. OS for normal, overweight and obese CRPC patients were 32.8, 47.6 and 43.4 months, respectively; *p* = 0.01. There was also a trend toward better PFS, but it was not statistically significant. Multivariate analysis found that obesity (BMI ≥ 30) was a separate protective factor for PFS in CSPC, while high-volume disease was a bad prognostic factor for OS in CRPC. A high Gleason score and ECOG-PS 2 were consistently associated with poor outcomes. *Conclusions*: Obesity has a phase-dependent prognostic influence in metastatic prostate cancer, providing a PFS advantage in CSPC and an OS benefit in CRPC. These results suggest that there may be an obesity paradox in people who are getting androgen receptor–targeted therapy. Further prospective studies are required to gain a better understanding of the biological reasons for this association.

## 1. Introduction

Obesity is a growing global health problem and has been increasingly recognized as a relevant factor influencing cancer incidence, progression, and treatment outcomes. Body mass index (BMI) is the most commonly used measure to classify body weight status, with normal weight defined as a BMI of 18.5–24.9 kg/m^2^, overweight as 25.0–29.9 kg/m^2^, and obesity as ≥30.0 kg/m^2^, according to World Health Organization criteria [1].

The prevalence of obesity among cancer patients is higher than that observed within the general population [2]. Obesity was associated with the increased risk of various cancers, such as breast, gastrointestinal, genitourinary, and hepatobiliary cancer [3,4]. The mechanisms underlying the relationship between obesity and cancer are unclear. Potential underlying mechanisms include direct interactions between peritumoral adipocytes and cancer cells, increased lipolysis in omental adipocytes resulting in free fatty acids, adipocyte–tumor cell cross-talk, and a diminished immune response due to chronic inflammation in obesity [5,6]. In addition, obesity leads to low-level systemic inflammation through various mechanisms, such as increased levels of interleukin-6 (IL-6) and tumor necrosis factor alpha (TNF-α) and an increase in M1-polarized macrophages [7]. When compared to the data, the relationship between obesity and treatment outcomes in cancer patients remains a subject of debate. A previous study indicated that being in the overweight and obesity range was not associated with inferior clinical outcomes in metastatic breast cancer, but low BMI was identified as an independent poor prognostic factor [8]. Another study reported that a high BMI served as a significant protective factor in patients with metastatic renal cell cancer [9]. This condition is described as a ‘paradoxical effect of obesity’, like the ‘reverse metabolism’ [10].

Cancer itself, along with medical treatments like surgery, chemotherapy and radiation therapy, all contribute to a catabolic state. In cancer patients, excess fat tissue may be effective in reducing mortality rates due to its high energy storage and strong immunological functions during this catabolic process. Patients who are overweight or obese may demonstrate greater treatment tolerance and an increased likelihood of completing full treatment courses. Sarcopenia is generally higher in older adults with cancer and is associated with higher chemotherapy-related toxicity and shorter survival outcomes [11]. Prostate cancer patients are predominantly elderly patients. Prostate cancer patients tend to be vulnerable to adverse outcomes due to both age-related and androgen deprivation therapy-induced sarcopenia and cancer-related cachexia [12]. Prostate cancer occurs more frequently in individuals with a high BMI [13]. Furthermore, obese prostate cancer patients undergoing curative treatment have a higher risk of cancer-related death [14]. However, data remains inconsistent in the metastatic context. While a significant association has been identified between BMI and metastatic prostate cancer using androgen receptor inhibitors such as enzalutamide and abiraterone, the relationship between BMI and survival outcomes is unclear [15,16,17]. The relationship between BMI and survival outcomes in both castration-resistant and castration-sensitive metastatic prostate cancer requires further investigation. Based on the hypothesis that a higher BMI may improve survival outcomes due to the paradoxical effect of obesity, we aimed to investigate the relationship between BMI and survival outcomes in patients with metastatic prostate cancer receiving androgen receptor pathway inhibitors.

## 2. Materials and Methods

The study included 167 patients with metastatic prostate cancer who were under follow-up at the Necmettin Erbakan University Faculty of Medicine, Department of Medical Oncology, and had a pathological diagnosis between 2015 and 2024. The research was conducted using a retrospective cohort study design. Patients in the castration-resistant and castration-sensitive stages were included. Patients with prostate cancer who experience recurrence after treatment of localized disease or develop metastases and have testosterone levels above 50 ng/dL are defined as having castration-sensitive prostate cancer (CSPC). A patient with biochemical and radiological progression while the serum testosterone level is below 50 ng/dL or 1.7 nmol/L is defined as having castration-resistant prostate cancer (CRPC) [18]. Disease volume was defined according to the CHAARTED trial criteria. High-volume disease was defined as the presence of visceral metastases and/or ≥4 bone metastases, with at least one metastatic lesion located outside the vertebral bodies and pelvis, whereas low-volume disease was defined as the absence of these features [19]. Risk status was classified based on the LATITUDE trial criteria. High-risk disease was defined by the presence of at least two of the following factors: Gleason score ≥ 8, presence of ≥3 bone metastases, or measurable visceral metastases. Patients not meeting these criteria were classified as having low-risk disease [20].

**Figure 1:**
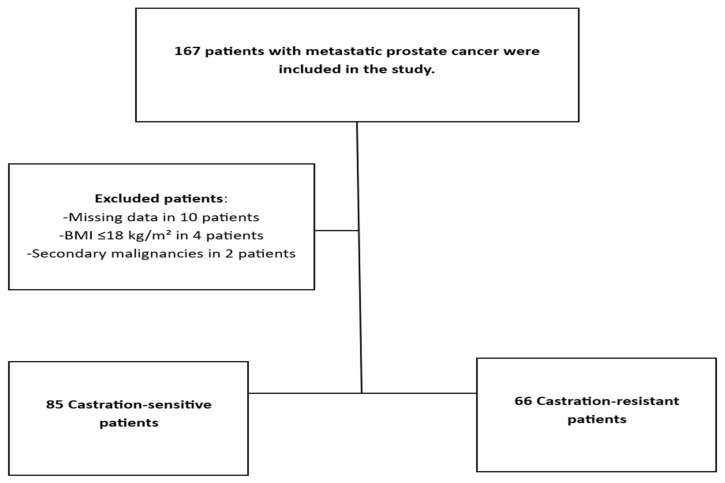
Study Flow Diagram.

Patients were stratified according to their clinical condition when first diagnosed, with a specific focus on whether they had Castration-Sensitive Prostate Cancer or Castration-Resistant Prostate Cancer (CRPC). Patients were excluded based on missing data, malnutrition (BMI < 18.5 kg/m^2^) and secondary malignancy, resulting in exclusions of 10, 4 and 2 patients, respectively (Figure 1).

The BMI was determined using height and weight measurements taken from patients prior to starting their oncological medical treatment. BMI was evaluated both as a continuous and a categorical variable. In addition, according to the World Health Organization’s adult BMI classification, patients were divided into three groups as normal weight (group 1) 18.5–24.9 kg/m^2^, overweight (group 2) 25–29.9 kg/m^2^, and obese (group 3) ≥30 kg/m^2^ [21]. Between these groups, age (≤70 vs. >70), Eastern Cooperative Oncology Group performance score (ECOG-PS 0–1 vs. 2), comorbidity (yes vs. no), statin use (yes vs. no), Gleason score (≤8 vs. >8), primary surgery (yes vs. no), volume status (high vs. low), risk status (high vs. low), de novo metastatic disease (yes vs. no), and androgen deprivation therapy (medical vs. surgical) were compared. Data is retrieved from clinical records and the hospital electronic health information system. This study was conducted with the formal approval of the local ethics committee (Approval no: 2025/6091).

Overall survival (OS) was defined as the time from the date of diagnosis to the date of death. Progression-free survival (PFS) was defined as the time from the start of androgen receptor pathway inhibitor therapy to the start date of disease progression.

### Statistical Analysis

All analyses were performed using the SPSS version 22.0 statistical software package (version 22.0, SPSS Inc., Chicago, IL, USA). Descriptive statistics were calculated as proportions and medians. The independent *t*-test was used to compare normally distributed data, while the Mann–Whitney U test was applied to non-normally distributed variables. Data were presented as mean ± standard deviation (SD) and median (min–max). The chi-square test was used to compare categorical variables between groups. The clinical endpoints were PFS and OS. Survival analysis was conducted using the Kaplan–Meier method. The log-rank test was used to compare survival between groups. A Cox regression model was used to identify risk factors for OS and PFS.

## 3. Results

The study included 85 patients with castration-sensitive and 66 patients with castration-resistant prostate cancer. The median follow-up time was 38 months. The median age was 70.7 years (49–91). The demographic data of the castration-sensitive and castration-resistant groups are presented in Table 1. There was no significant difference between BMI groups in terms of age, ECOG-PS, statin use, comorbidity, Gleason score, primary surgery, de novo metastatic status, disease volume, disease risk, and ADT status in the castration-sensitive and castration-resistant groups (Table 2).

**Figure 2:**
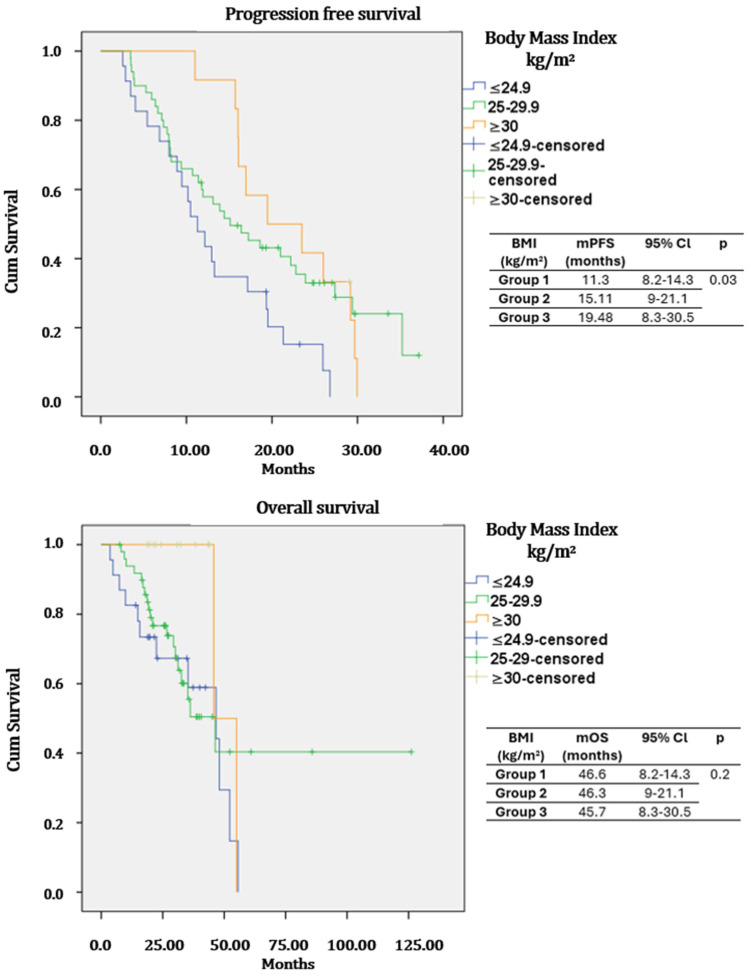
Kaplan–Meier curves of overall survival (OS) and progression-free survival (PFS) by body mass index (BMI) in the metastatic castration-sensitive group.

**Figure 3:**
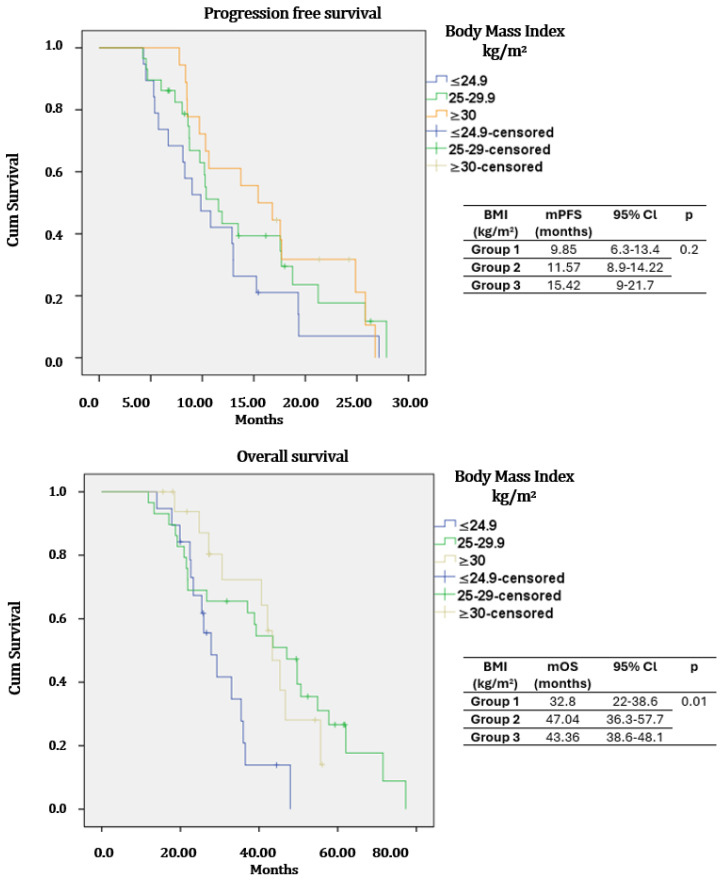
Kaplan–Meier curves of overall survival (OS) and progression-free survival (PFS) by body mass index (BMI) in the metastatic castration-resistant group.

In the study population, mPFS was 11.79 months (95%Cl: 8.33–15.24), and mOS was 29.01 months (95%Cl: 25.82–32.19). In the entire study group, mOS was 36.4 months (95%CI: 28.2–44.5), 46.8 months (95%CI: 38.6–54.9), and 44.5 months (95%CI: 39.2–49.7) for normal weight (Group 1), overweight (Group 2), and obese (Group 3) patients, respectively, with this difference favoring overweight and obese patients being statistically significant (*p* = 0.04). In the CSPC group, the mPFS was 11.3 months (95%Cl: 8.2–14.3) for normal weight, 15.11 months (95%Cl: 9–21.1) for overweight and 19.48 months (95%Cl: 8.3–30.5) for obese patients (*p* = 0.03). The mOS of castration-sensitive patients was 46.6 months (95%Cl: 23–70.3) for normal weight, 46.3 months (95%Cl: 29.4–63.1) for overweight and 45.7 months (NR) for obese patients (*p* = 0.2) (Figure 2). The mPFS of castration-resistant patients was 9.85 months (95%Cl: 6.3–13.4) for normal weight, 11.57 months (95%Cl: 8.9–14.22) for overweight, and 15.42 months (95%Cl: 9–21.7) for obese patients (*p* = 0.2). The mOS of castration-resistant patients was 32.8 months (95%Cl: 22–38.6) in group 1, 47.6 (95%Cl: 36.3–57.7) months in group 2, and 43.36 months (95%Cl: 38.6–48.1) in group 3 (*p* = 0.01) (Figure 3).

In the castration-sensitive group, ECOG-PS (*p* = 0.03), BMI (*p* = 0.036), and disease volume (*p* = 0.004) were statistically significant for PFS in the univariate analysis. In the multivariate analysis, a BMI ≥ 30 was identified as a favorable prognostic marker for PFS (HR: 0.67, 95%CI: 0.20–0.99, *p* = 0.04). Additionally, having low-volume disease (HR: 0.54, 95%CI: 0.30–0.85, *p* = 0.01) was also favorable for PFS. However, having an ECOG-PS of 2 (HR: 3.06, 95%CI: 1.28–7.29, *p* = 0.011) was unfavorable for PFS. In the castration-sensitive group, Gleason score (*p* = 0.02) and disease volume status (*p* = 0.028) were statistically significant for OS in the univariate analysis. In the multivariate analysis, a Gleason score of 9–10 was found to be an unfavorable prognostic marker for OS (HR: 1.50, 95%CI: 1.01–2.71, *p* = 0.03). Additionally, an ECOG-PS of 2 (HR: 1.50, 95%CI: 1.08–5.24, *p* = 0.03) was unfavorable for OS. However, having low disease volume (HR: 0.75, 95%CI: 0.41–0.96, *p* = 0.04) was favorable for OS (Table 3).

In the castration-resistant group, Gleason score (*p* = 0.002) and disease volume (*p* = 0.01) were statistically significant for PFS in the univariate analysis. In the multivariate analysis, a Gleason score of 9–10 (HR: 2.60, 95%CI: 1.27–5.31, *p* = 0.008) was an unfavorable prognostic factor for PFS. In the castration-resistant group, BMI (*p* = 0.02) and disease volume status (*p* = 0.028) were statistically significant for OS in the univariate analysis. In the multivariate analysis, high-volume disease was identified as an unfavorable prognostic marker for OS (HR: 2.47, 95%CI: 1.40–2.98, *p* = 0.019) (Table 4).

## 4. Discussion

In castration-sensitive patients, PFS was significantly prolonged in obese patients compared to those of normal weight or overweight status; no statistically significant difference was observed in OS. In the castration-resistant group, OS was significantly greater in obese and overweight patients, with a non-significant trend toward improved PFS. These findings are consistent with the increasingly discussed “obesity paradox” in the literature.

Research on how obesity affects cancer prognosis has often produced conflicting results. Obesity is considered a risk factor for the development of many solid tumors through mechanisms such as chronic inflammation, insulin resistance, adipokine imbalance, and hormonal alterations [3,22]. In prostate cancer, obesity has been associated with both incidence and progression [13,23]. However, in the metastatic cases, particularly among patients treated with agents targeting the androgen receptor pathway, obesity’s impact on survival is more complicated. Pan et al. [17] reported that in CRPC patients receiving abiraterone, a higher BMI, when evaluated together with albumin levels, was associated with longer survival. Fischer et al. [16] demonstrated that changes in body composition during enzalutamide and abiraterone therapy may influence survival outcomes. Similarly, in the BONENZA study reported by Buffoni et al. [15], it was noted that increased adipose tissue in obese patients undergoing androgen deprivation therapy may positively affect treatment tolerance.

In our study, the observation of a PFS advantage in obese patients within the castration-sensitive group suggests that obesity may be associated with response to hormonal therapy. Androgen deprivation therapy (ADT) often leads to sarcopenia and cachexia [14]. During this process, adipose tissue in obese patients may function as an energy reserve, modulate the inflammatory response, and enhance treatment tolerance, which may represent underlying mechanisms. Additionally, steroid precursors derived from adipose tissue may influence the pharmacodynamics of abiraterone. In the castration-resistant group, the OS advantage observed in obese patients may be explained by the “reverse metabolism” phenomenon, wherein preservation of energy reserves confers benefit in advanced-stage cancers [24]. A recent study demonstrated that visceral adipose tissue reduces chemotherapy toxicity in obese prostate cancer patients, improving treatment adherence and providing a survival advantage [25]. These findings support the obesity paradox observed in our study and strengthen the hypothesis that high BMI values may serve as a protective energy reserve and pharmacokinetic shield in the metastatic process. The observed phase-dependent differential effect of obesity can be explained by several biological mechanisms. In the CSPC stage, obesity may delay progression by modulating androgen synthesis via steroid precursors originating from adipose tissue and affecting the pharmacokinetics of androgen receptor pathway inhibitors (ARPIs). In contrast, in the CRPC stage, where the disease is a more advanced catabolic process, a high BMI acts as a “metabolic reserve”, protecting against cancer cachexia and providing an OS advantage. Similarly, a protective effect of high BMI has been reported in metastatic renal cancer, and low BMI has been identified as a poor prognostic factor in metastatic breast cancer [10,11]. The number of patients in each subgroup within the CSPC group is relatively small; therefore, the obtained HRs should be interpreted carefully.

In the castration-sensitive group, an ECOG performance score of 2 was identified as an adverse prognostic factor for both PFS and OS, a finding consistent with previous meta-analyses [13]. As expected, high Gleason score and high disease volume were also unfavorable prognostic variables. In contrast, obesity may have provided a more pronounced survival advantage, particularly in patients with low-volume metastatic disease. This suggests heterogeneous biological effects within the obesity–inflammation axis [5].

A key strength of our study is the separate analysis of both castration-sensitive and castration-resistant patient groups. However, the retrospective design and limited sample size may have introduced selection bias, thereby restricting the generalizability of the findings. Additionally, BMI alone may not fully reflect body composition; evaluation of variables such as muscle-to-fat ratio would provide more comprehensive insights into conditions like sarcopenic obesity. Another limitation of this study is that patients receiving abiraterone and enzalutamide were analyzed together. No separate adjustments were made for treatment durations, drug sequencing or agent-specific pharmacological differences. Additionally, our study did not account for patients’ history of diabetes or blood glucose levels; given that hyperglycemia has been linked to increased disease severity and poorer outcomes in cancer, the absence of this metabolic data represents a limitation of our analysis.

## 5. Conclusions

In conclusion, this study demonstrates that the impact of obesity on survival in patients with metastatic prostate cancer differs by treatment phase. Obesity may be a prognostic factor for PFS in castration-sensitive patients and for OS in castration-resistant patients. These findings suggest the obesity paradox may also be applicable to prostate cancer patients treated with androgen receptor inhibitors. However, large-scale prospective studies are needed to elucidate the underlying biological mechanisms of this association.

## Figures and Tables

**Table 1:** General characteristics of castration-sensitive and castration-resistant groups.

Features		Castration-Sensitive Group (*n* = 85)	Castration-Resistant Group (*n* = 66)
Age (years)	≤70	35 (41.2%)	33 (50%)
	>70	50 (51.8%)	33 (50%)
Body Mass Index (kg/m^2^)	<25	23 (27.1%)	4 (6.1%)
	25–29.9	50 (58.8%)	36 (54.5%)
	≥30	12 (14.1%)	26 (39.4%)
ECOG-PS	0	14 (16.5%)	19 (28.8%)
	1	51 (60%)	29 (43.9%)
	≥2	20 (23.5%)	18 (27.3%)
Statin use	No	66 (77.6%)	49 (74.2%)
	Yes	19 (22.4%)	17 (25.8%)
Comorbidity	No	39 (45.9%)	30 (45.5%)
	Yes	46 (54.1%)	36 (54.5%)
Gleason score	≤8	20 (23.5%)	19 (28.8%)
	9–10	65 (76.5%)	47 (71.2%)
Primary surgery	No	74 (87.1%)	56 (84.8%)
	Yes	11 (12.9%)	10 (15.2%)
De novo metastatic	No	26 (30.6%)	23 (34.8%)
	Yes	59 (69.4%)	43 (65.2%)
Disease volume *	Low	22 (25.9%)	9 (13.6%)
	High	63 (74.1%)	57 (86.4%)
Disease risk **	Low risk	21 (24.7%)	9 (13.6%)
	High risk	64 (75.3%)	57 (86.4%)
Medical treatment	Abiraterone acetate	46 (54.1%)	33 (50%)
	Enzalutamide	39 (45.9%)	33 (50%)
Androgen deprivation therapy (ADT)	Medical	83 (97.6%)	63 (95.5%)
	Surgery	2 (2.4%)	3 (4.5%)

* CHAARTED trial; ** LATITUDE trial.

**Table 2:** Comparison of patient characteristics according to Body Mass Index in castration-resistant and castration-sensitive groups.

Features		Castration-Sensitive Group				Castration-Resistant Group			
		Body Mass Index (kg/m^2^)							
		Group 1 (≤24.9)	Group 2 (25–29.9)	Group 3 (≥30)	*p*	Group 1 (≤24.9)	Group 2 (25–29.9)	Group 3 (≥30)	*p*
Age (years)	≤70	11 (47.8%)	14 (34%)	7 (58.3%)	0.23	9 (47.4%)	14 (48.3%)	10 (55.6%)	0.85
	>70	12 (52.2%)	33 (66%)	5 (41.7%)		10 (52.6%)	15 (51.7%)	8 (44.4%)	
ECOG-PS	0–1	19(82.6%)	35 (70%)	11(91.7%)	0.075	11(57.9%)	20(68.9%)	9 (50%)	0.62
	2	4 (17.4%)	15 (30%)	1 (8.3%)		8 (42.1%)	9 (31%)	9 (50%)	
Statin use	No	18 (78.3%)	38 (76%)	10 (83.3%)	0.85	14 (73.7%)	21 (72.4%)	14 (77.8%)	0.91
	Yes	5 (21.7%)	12 (24%)	2 (16.7%)		5 (26.3%)	8 (27.6%)	4 (22.2%)	
Comorbidity	No	13 (56.5%)	21 (42%)	5 (41.7%)	0.48	10 (52.6%)	12 (41.4%)	8 (44.4%)	0.74
	Yes	10 (43.5%)	29 (58%)	7 (58.3%)		9 (47.4%)	17 (58.6%)	10 (56.6%)	
Gleason score	≤8	5 (21.7%)	12 (24%)	3 (25%)	0.64	5 (26.3%)	10 (34.5%)	4 (22.2%)	0.64
	9–10	18 (78.3%)	38 (76%)	9 (75%)		14 (73.7%)	19 (65.5%)	14 (77.8%)	
Primary surgery	No	22 (95.7%)	43 (86%)	9 (75%)	0.21	17 (89.5%)	25 (86.2%)	14 (77.8%)	0.58
	Yes	1 (4.3%)	7 (14%)	3 (25%)		2 (10.5%)	4 (13.8%)	4 (22.2%)	
De novo metastatic	No	5 (21.7%)	13 (26%)	8 (66.7%)	0.13	5 (26.3%)	11 (37.9%)	17 (38.9%)	0.65
	Yes	18 (78.3%)	37 (74%)	4 (33.3%)		14 (73.7%)	18 (62.1%)	11 (61.1%)	
Disease volume *	Low	5 (21.7%)	10 (20%)	7 (58.3%)	0.20	3 (15.8%)	4 (13.8%)	2 (11.1%)	0.91
	High	18 (78.3%)	40 (80%)	5 (41.7%)		16 (84.2%)	25 (86.2%)	16 (88.9%)	
Disease risk **	Low risk	4 (17.4%)	10 (20%)	7 (58.3%)	0.14	3 (15.8%)	4 (13.8%)	2 (11.1%)	0.91
	High risk	19 (82.6%)	40 (80%)	5 (41.7%)		16 (84.2%)	25 (86.2%)	16 (88.9%)	
Medical treatment	Abiraterone acetate	13 (56.5%)	31 (92%)	2 (16.7%)	0.18	11 (57.9%)	13 (44.8%)	9 (50%)	0.67
	Enzalutamide	10 (43.5%)	19 (38%)	10 (83.3%)		8 (42.1%)	16 (55.2%)	9 (50%)	
Androgen deprivation therapy	Medical	0 (0%)	2 (4%)	0 (0%)	0.78	1 (5.3%)	1 (3.4%)	1 (5.6%)	0.84
	Surgery	23 (100%)	48 (96%)	12 (100%)		18 (94.7%)	28 (96.6%)	17 (94.4%)	

* CHAARTED trial; ** LATITUDE trial.

**Table 3:** Evaluation of predictive factors for overall survival and progression-free survival in the castration-sensitive group by univariate and multivariate analysis.

**Progression-Free Survival**		**Castration-Sensitive Group**					
		**Univariate**			**Multivariate**		
		**Hazard Ratio**	**95% Confidence Interval**	***p***	**Hazard Ratio**	**95% Confidence Interval**	***p***
Age	≤70 vs. >70	0.68	0.41–1.12	0.13	-	-	-
ECOG-PS	0	Reference		0.08	Reference		0.029
	1	1.71	0.79–3.66	0.16	2.73	1.22–6.10	0.014
	2	2.52	1.09–5.83	0.03	3.06	1.28–7.29	0.011
BMI	≤24.9	Reference		0.036	Reference		0.09
	25–29.9	0.50	0.29–0.88	0.017	0.70	0.22–1.12	0.20
	≥30	0.64	0.31–0.97	0.043	0.67	0.20–0.99	0.04
Statin use	No vs. Yes	0.71	0.41–1.24	0.24	-	-	-
Comorbidity	No vs. Yes	0.72	0.43–1.16	0.17	-	-	-
Gleason score	≤8 vs. 9–10	1.38	0.79–2.41	0.25	-	-	-
Disease volume *	Low vs. High	0.41	0.23–0.75	0.004	0.54	0.30–0.85	0.01
De novo metastatic	No vs. Yes	1.47	0.89–2.44	0.13	-	-	-
**Overall Survival**		**Castration-Sensitive Group**					
		**Univariate**			**Multivariate**		
		**Hazard Ratio**	**95% Confidence Interval**	***p***	**Hazard Ratio**	**95% Confidence Interval**	***p***
Age	≤70 vs. >70	0.70	0.34–1.43	0.33	-	-	-
ECOG-PS	0	Reference		0.08	Reference		0.07
	1	1.88	0.43–8.18	0.39	2.70	0.62–4.87	0.17
	2	2.85	0.86–7.23	0.07	3.90	1.08–5.24	0.03
BMI	≤24.9	Reference		0.24	Reference		0.18
	25–29.9	0.73	0.35–1.52	0.41	0.64	0.30–1.38	0.26
	≥30	0.68	0.35–1.26	0.09	0.62	0.27–1.21	0.08
Statin use	No vs. Yes	0.78	0.37–1.65	0.52	-	-	-
Comorbidity	No vs. Yes	0.78	0.38–1.59	0.5	-	-	-
Gleason score	≤8 vs. 9–10	1.52	1.09–2.24	0.02	1.50	1.01–2.71	0.03
Disease volume *	Low vs. High	0.48	0.26–0.89	0.028	0.75	0.41–0.96	0.04
De novo metastatic	No vs. Yes	1.22	0.78–1.88	0.21	-	-	-

* CHAARTED trial.

**Table 4:** Evaluation of predictive factors for overall survival and progression-free survival in the castration-resistance group by univariate and multivariate analysis.

**Progression-Free Survival**		**Castration-Resistance Group**					
		**Univariate**			**Multivariate**		
		**Hazard Ratio**	**95% Confidence Interval**	***p***	**Hazard Ratio**	**95% Confidence Interval**	***p***
Age	≤70 vs. >70	1.04	0.58–1.71	0.98	-	-	-
ECOG-PS	0	Reference		0.18	-	-	-
	1	0.37	0.18–1.64	0.19	-	-	-
	2	1.31	0.75–2.30	0.33	-	-	-
BMI	≤24.9	Reference		0.18	Reference		0.2
	25–29.9	0.77	0.41–1.47	0.44	0.90	0.55–190	0.81
	≥30	0.52	0.26–1.04	0.06	0.60	0.15–1.21	0.15
Statin use	No vs. Yes	0.75	0.55–1.42	0.74	-	-	-
Comorbidity	No vs. Yes	0.48	0.30–1.25	0.55	-	-	-
Gleason score	≤8 vs. 9–10	0.33	0.16–0.66	0.002	2.60	1.27–5.31	0.008
Disease volume *	Low vs. High	0.55	0.35–0.89	0.01	2.25	0.73–6.97	0.15
De novo metastatic	No vs. Yes	0.64	0.35–1.17	0.15	-	-	-
**Overall Survival**		**Castration-Resistant Group**					
		**Univariate**			**Multivariate**		
		**Hazard Ratio**	**95% Confidence Interval**	***p***	**Hazard Ratio**	**95% Confidence Interval**	***p***
Age	≤70 vs. >70	0.56	0.30–1.02	0.06	1.45	0.70–2.99	0.32
ECOG-PS	0	Reference		0.06	Reference		0.54
	1	3.50	0.47–6.05	0.22	1.11	0.70–4.42	0.86
	2	6.32	0.82–8.27	0.07	1.21	0.81–4.9	0.87
BMI	≤24.9	Reference		0.33	Reference		0.71
	25–29.9	0.67	0.34–1.32	0.02	0.79	0.40–1.56	0.50
	≥30	0.56	0.25–1.27	0.16	0.71	0.25–1.72	0.45
Statin use	No vs. Yes	0.78	0.55–1.20	0.48	-	-	-
Comorbidity	No vs. Yes	0.85	0.64–1.48	0.69	-	-	-
Gleason score	≤8 vs. 9–10	0.52	0.26–1.00	0.052	1.71	1.40–2.98	0.06
Disease volume *	Low vs. High	0.42	0.22–0.73	0.013	2.47	1.63–6.70	0.019
De novo metastatic	No vs. Yes	0.45	0.15–1.11	0.36	-	-	-

* CHAARTED trial.

## Data Availability

The data that support the findings of this study are available on request from the corresponding author. The data are not publicly available due to privacy or ethical restrictions.

## Abbreviations

The following abbreviations are used in this manuscript:

CSPC	Castration-sensitive prostate cancer
CRPC	Castration-resistant prostate cancer
BMI	Body Mass İndex
PFS	progression-free survival
OS	overall survival
ECOG-PS	Eastern Cooperative Oncology Group performance score
ADT	Androgen deprivation therapy
